# Transcriptome-Based Identification and Functional Characterization of NAC Transcription Factors Responsive to Drought Stress in *Capsicum annuum* L.

**DOI:** 10.3389/fgene.2021.743902

**Published:** 2021-10-22

**Authors:** Dionis Borràs, Lorenzo Barchi, Karina Schulz, Andrea Moglia, Alberto Acquadro, Iman Kamranfar, Salma Balazadeh, Sergio Lanteri

**Affiliations:** ^1^ Department of Agricultural, Forest and Food Sciences, Plant Genetics and Breeding, University of Torino, Turin, Italy; ^2^ Max Planck Institute of Molecular Plant Physiology, Potsdam, Germany; ^3^ Department Molecular Biology, Institute of Biochemistry and Biology, University of Potsdam, Potsdam, Germany; ^4^ Plant Sciences and Natural Products, Institute of Biology Leiden (IBL), Leiden University, Leiden, Netherlands

**Keywords:** bell pepper, transcriptome, drought tolerance, NAC, VIGS, functional characterization

## Abstract

*Capsicum annuum* L. is one of the most cultivated Solanaceae species, and in the open field, water limitation leading to drought stress affects its fruit quality, fruit setting, fruit size and ultimately yield. We identified stage-specific and a common core set of differentially expressed genes, following RNA-seq transcriptome analyses of a breeding line subjected to acute drought stress followed by recovery (rewatering), at three stages of plant development. Among them, two NAC transcription factor (TF) genes, i.e., CaNAC072 and CaNAC104, were always upregulated after drought stress and downregulated after recovery. The two TF proteins were observed to be localized in the nucleus following their transient expression in *Nicotiana benthamiana* leaves. The expression of the two NACs was also induced by NaCl, polyethylene glycol (PEG) and abscisic acid (ABA) treatments, suggesting that *CaNAC072* is an early, while *CaNAC104* is a late abiotic stress-responsive gene. Virus-induced gene silencing (VIGS) of *CaNAC104* did not affect the pepper plantlet’s tolerance to drought stress, while VIGS of *CaNAC072* increased drought tolerance. Heterologous expression of *CaNAC072* in *Arabidopsis thaliana* as well as in plants mutated for its homolog *ANAC072* did not increase drought stress tolerance. This highlights a different role of the two NAC homologs in the two species. Here, we discuss the complex role of NACs as transcriptional switches in the response to drought stress in bell pepper.

## Introduction

Drought stress is one of the key limiting factors affecting plant growth, development and survival, with a substantial impact on crop yield (Sinclair, 2011; Boyer et al., 2013). The lack of water, due to rising temperatures and changes in precipitation patterns, represents a serious problem in many parts of the world, and it is expected to become a cause of food shortage and malnutrition with the increase in population and food demand (Newton et al., 2011; Fitton et al., 2019). Plants have evolved various molecular mechanisms to adjust their growth to limited water availability, and the elucidation of such mechanisms is essential for implementing breeding strategies aimed at developing crop varieties more resilient and capable to deal with water shortage while maintaining yield (Osakabe et al., 2014; Iovieno et al., 2016). Bell pepper (*Capsicum annuum* L.), also known as sweet pepper, is one of the most widely cultivated solanaceous vegetables (FAOSTAT, 2018), and particularly at flowering, water deficit causes flower dropping which reduces the setting and size of the fruits, and alters their biochemical composition (Borras et al., 2020).

Comparative transcriptomic analyses have proven to be a valuable approach for identifying key genes controlling the response to low water availability (Seki et al., 2002) in plants such as Arabidopsis (Zhang et al., 2019), rice (Chung et al., 2018), maize (Kakumanu et al., 2012), potato (Sprenger et al., 2018), and tomato (Lee et al., 2018). However, in C. *annuum*, studies on transcriptional changes occurring in response to water shortage are to date rather limited (Zinselmeier et al., 2002; Ji et al., 2010; Sharoni et al., 2012; Lee and Choi, 2013).

Previous studies in plants have highlighted that transcription factors (TFs) play a crucial role as molecular regulators to activate or inhibit the expression of stress-related genes and promote adaptation to stressful environments. Important abiotic stress-responsive TF families are AP2/ERF, DREB, bZIP, AREB/ABF, MYB, bHLH, WRKY and NAC (Nakashima et al., 2012; Singh and Laxmi, 2015; Gamboa-Tuz et al., 2018; Mittal et al., 2018; Mohanta et al., 2020). With respect to NACs, several members of this family have been identified and functionally characterized in crops, among which is the solanaceous species tomato (Yang et al., 2011; Han et al., 2012; Ma et al., 2013; Thirumalaikumar et al., 2018; Devkar et al., 2020). The typical NAC protein is characterized by a highly conserved DNA-binding NAC domain at its N-terminus and a divergent transcription regulatory region (TRR) at its C-terminus (Mao et al., 2007).

Whole-genome sequences of both peppers and closely related species have recently been made available (Kim et al., 2014; Qin et al., 2014; Kang et al., 2016; Ahn et al., 2018; Acquadro et al., 2020), and a comprehensive analysis of the NAC gene family has been performed. A whole of 104 *CaNAC* genes, of which 24 are exclusive to the Solanaceae family, were identified and found to be mostly located on chromosomes 1, 2, 3, and 6 (Diao et al., 2018). However, few NAC genes have been functionally characterized in C. *annuum* with respect to their function in stress responses. Recently, Zhang et al. (2020) isolated and functionally characterized CaNAC035, a positive regulator of abiotic stress tolerance acting through multiple signaling pathways. The involvement in the response to abiotic stress tolerance has also been demonstrated for CaNAC064 (Hou et al., 2020) and CaNAC2 (Guo et al., 2015).

In this study, we employed RNA-sequencing to identify differentially expressed genes (DEGs) in a *C. annuum* inbred line subjected to acute drought stress, and recovery from it, at different developmental stages. Common core sets and plant developmental stage-specific DEGs were identified. Among them, two members of the NAC family (namely *CaNAC072* and *CaNAC104*) were significantly up-regulated transcriptionally following acute drought stress, and down-regulated after recovery across all developmental stages, and were thus selected for further functional characterization. Transient expression in *Nicotiana benthamiana* leaves revealed a nuclear localization of CaNAC072 and CaNAC104; however, a different time of transcriptional activation of the two pepper NAC genes was detected following NaCl, PEG or ABA treatments.

Both NACs were also functionally characterized by Virus-Induced Gene Silencing (VIGS) in pepper plantlets, and CaNAC072 was further assessed in three *Arabidopsis thaliana* genetic backgrounds.

## Material and Methods

### Drought Stress Treatments, RNA Extraction and Sequencing

Seeds of a yellow-fruited breeding line of *Capsicum annuum* (Cuneo CCu07), selected at the DISAFA (University of Turin), were placed on filter paper soaked with sterilized tap water. Petri dishes were placed in a growth chamber at 350 μmol photons m^−2^ s^−1^, 50% relative humidity, 26–24°C and a 16−8 h light-dark regime. After germination, seedlings were transferred to individual plastic pots filled with peat and grown for about 15 days. Before transfer to the greenhouse, the plantlets were transplanted in 5 L pots filled with a mixture of peat and perlite at a ratio of 1:1 by volume and grown following standard horticultural practices.

Groups of 15 plants were irrigated up to the soil water retention, and grown up to: 1) the production of five true leaves (stage 1); 2) the production of the third flower (stage 2); 3) the setting of the first fruit (stage 3). Ten plants at each developmental stage were subjected to drought stress by stopping irrigation, while irrigation was maintained on five other plants (Controls). The effect of drought stress was assessed by evaluating stomatal conductance (g_s_) through a Leaf Porometer Model SC-1 (Meter Group) on five fully expanded and randomly chosen leaves of each plant. Each measurement was replicated three times in each plant. After drought stress, a group of five plants was recovered following re-watering up to the soil water retention, until they reached the same g_s_ as control plants.

At each stage of plant development, RNA was extracted using the NucleoSpin RNA kit (Macherey-Nagel) from three leaves of three control plants as well as three plants subjected to drought stress, and three plants subjected to drought stress followed by recovery, chosen randomly. The RNAs were treated with RNase-free DNase I at room temperature to remove contaminant DNA.

Prior to library construction, an Agilent 2,100 Bioanalyzer (Agilent, CA, United States) was used to confirm the quality and quantity of RNA such that the 28S/18 S rRNA ratio was >1.5 and the RNA integrity number >7. For each sample, mRNA was fragmented randomly by adding fragmentation buffer, then the cDNA was synthesized by using mRNA template and random hexamer primers, after which a custom second-strand synthesis buffer (Illumina), dNTPs, RNase H and DNA polymerase I were added to initiate the second-strand synthesis. After terminal-end repair and ligation of sequencing adaptors, the double-stranded cDNA library was completed through size selection and PCR enrichment. Libraries were sequenced on an Illumina NovaSeq 6,000 platform at Novogene, and 150 bp paired-end reads were generated. Raw reads are publicly available in the NCBI Sequence Read Archive under Bioproject PRJNA668245.

### Identification of Differentially Expressed Genes and Enrichment Analysis

Raw reads obtained from the Illumina NovaSeq 6,000 platform were processed for standard quality controls (QC) using FastQC (https://www.bioinformatics.babraham.ac.uk/projects/fastqc/). Then they were cleaned with Scythe (v0.994, https://github.com/vsbuffalo/scythe) for removing contaminant residual adapters, and Sickle (v1.33, https://github.com/najoshi/sickle) to remove reads with poor quality ends (Q < 30). The cleaned reads were mapped to the reference CM334 pepper genome (Kim et al., 2017) with Hisat2 (Kim et al., 2015). Data quality analysis was conducted by visualizing results of principal component analysis (PCA). The counts of mapped reads per gene per sample were obtained with Stringtie (Pertea et al., 2015). Then, the DESeq2 R package was applied to identify differentially expressed genes (DEGs) (Love et al., 2014) at a False Discovery Rate (FDR) threshold of 0.05. The genes with an absolute log2 fold change ≥1 were considered as DEGs.

Functional enrichment analyses of Gene Ontology (GO) annotation was performed to identify which DEGs were significantly enriched in GO terms. GO enrichment analysis was conducted using AGRIGOv2, using the available annotation of the previously published sequence of the inbred line CCu07 (Acquadro et al., 2020).

### NAC Gene Prediction and Transcription Factor Identification

The Hidden Markov Model (HMM) profile of the NAC domain (PF02365), downloaded from the Protein families (Pfam) database (El-Gebali et al., 2019), was used for identifying NAC genes in the pepper CM334 genome (Kim et al., 2017) using HMMER (V 3.2.1). Positive matches were manually checked to confirm the presence of NAC domains, using InterProScan (Mitchell et al., 2019). Transcription factors in the reference genome CM334 were identified following a BlastP analysis against the Plant Transcription Factor Database (http://planttfdb.gao-lab.org/). To identify Arabidopsis orthologs of two NACs, which were always upregulated after drought stress and downregulated after recovery (i.e., CA.PGAv.1.6.scaffold213.1 and CA.PGAv.1.6.scaffold1156.46), a multiple alignment with the entire Arabidopsis NAC family was carried out by applying Clustal Omega (1.2.4) (Madeira et al., 2019). A further Pairwise Sequence Alignment with Emboss Needle (Madeira et al., 2019), for both pepper NACs with the closest Arabidopsis gene was used to retrieve identity and similarity percentage.

### Subcellular Localization

Subcellular localization of CaNAC072 and CaNAC104 was predicted by identifying Nuclear Localization Signal (NLS) sequences using the Localizer software (http://localizer.csiro.au/).

The coding sequences (without stop codon) of CaNAC072 and CaNAC104 were amplified from CCu07 cDNA by using primers CaNAC072-F, CaNAC072-R, CaNAC104-F and CaNAC104-R (Supplementary Table S1). PCR was performed with Phusion High-Fidelity DNA polymerase (Thermo Fisher Scientific) and the amplicon sequences were validated (Eurofins MWG Operon). Amplicons were cloned into pDONR207 vector using BP clonase (Invitrogen). The sequence-confirmed entry vector was recombined into pK7FWG2, containing kanamycin as selection marker (Karimi et al., 2002), using LR reaction mix II (Invitrogen). The recombined plasmids (35S:CaNAC072-GFP and 35S:CaNAC104-GFP) were transformed into *Agrobacterium tumefaciens* (strain GV3101) and then used for infiltration of *Nicotiana benthamiana* leaves (Senthil-Kumar and Mysore, 2014). GFP signals were analyzed 2 days after leaf infiltration using a Leica DM6000B/SP5 confocal laser-scanning microscope (Leica Microsystems, Wetzlar, Germany). The laser excitation wavelength was 488 nm, and the detection was set at 507 nm.

Further confirmation of nuclear signal was assessed with nuclear-specific DAPI (4,6-diamidino-2-phenylindole) staining. Transformed leaves were incubated for 30 min in darkness with a solution containing 10 mg L^−1^ DAPI. Afterwards, nuclear-specific signal was assessed by confocal laser-scanning microscopy as described by Sampathkumar and Wightman (2015).

### Stress and Hormonal Treatments

CCu07 seeds were germinated on full-strength Murashige-Skoog (MS) medium containing 2% (w/v) sucrose, and grown under a 24–26°C and a 16–8 h light-dark regime. Ten-day-old seedlings were transferred to MS liquid media containing 120 mM NaCl (Sigma-Aldrich), 10% (w/v) PEG 6000 (Sigma-Aldrich), or 100 μM ABA (Sigma-Aldrich), and treated for 2, 4, 6, 10 and 24 h. Stressors and hormones were omitted from control seedlings.

### Virus-Induced Gene Silencing

For the virus-induced gene silencing (VIGS) experiment, CCu07 seeds were sown directly into soil containing a mixture of peat and vermiculite (2:1, v/v), and plants grown in a phytotron at 450 μmol photons m^−2^s^−1^, 50% relative humidity, 21°C day/night under a 14 h day/10 h night regime.

VIGS was performed with vectors pTRV1 (pYL192) (Liu et al., 2002) and pTRV2 (pYL170) following the protocol described by Senthil-Kumar and Mysore, 2014. Briefly, VIGS constructs were designed with the SGN VIGS Tool (Fernandez-Pozo et al., 2015). The selected gene fragments (Supplementary Table S2) were PCR-amplified using primers VIGS_CaNAC072-F, VIGS_CaNAC072-R, and VIGS_CaNAC104-F, VIGS_CaNAC104-R (Supplementary Table S1) from CCu07 cDNA and cloned into pDONR207 using BP clonase (Invitrogen). The sequence-confirmed entry vector was recombined into pK7FWG2 using LR reaction mix II (Life Technologies). The plasmids pTRV1, pTRV2:CaNAC072, pTRV2:CaNAC104 as well as pTRV2:AtPDS (as a positive control for efficient VIGS) and empty vector pTRV2:00 were used to transform A. tumefaciens (strain GV3101), and the latter used for infecting cotyledons of 10 days-old pepper seedlings. After 12 days of infection, drought stress was performed for 7 and 9 days. Recovery treatments were performed by re-watering plants for 7 days. For assessing plant survival rate, plants were subjected to 9 days severe drought stress followed by recovery through re-watering. The percentage of surviving plants was determined after 7 days of recovery. The drought and recovery phenotypic analyses were assessed in 15 biological replicates per treatment.

### Identification of Pepper Homologous Genes in Arabidopsis and Plant Transformation

Pepper orthologs of Arabidopsis drought marker genes [i.e., *ANAC019* (AT1G52890), *ANAC055* (AT3G15500), *DREB2A* (AT5G05410), *DELLA/GAI* (AT1G14920), and *SnRK2* (AT3G50500)], were identified using PLAZA 4.5 (https://bioinformatics.psb.ugent.be/plaza/versions/plaza_v4_5_dicots/) and annotated using the Sol Genomics Database (https://solgenomics.net/).

The 35S:CaNAC072-GFP and 35S:CaNAC104-GFP constructs were inserted into *A. tumefaciens* (strain GV3101) and transformed into *A. thaliana* genotypes Columbia-0 (Col-0), *anac072* mutant (*rd26-2*, SALK_083,756) and *anac019/anac055/anac072* triple mutant backgrounds using the floral dip method (Clough and Bent, 1998). NAC T-DNA insertion lines (*anac019*: SALK_096,295, *anac055*: SALK_014331, and *anac072*: SALK_083756) were obtained from NASC (http://arabidopsis.info). Homozygous knockout plants were identified by PCR-based genotyping, qRT-PCR, and end-point PCR. The triple NAC mutant was generated by crossing. Plants were grown at 22°C under long-day (LD) condition, i.e., 16 h light/8 h dark. Seeds of T2 plants were harvested.

From T1 transgenic plants showing a 3:1 segregation ratio when grown on MS plates supplemented with the same antibiotic. Ten-day-old seedlings were transferred to soil [potting and quartz sand; 2:1 (v/v)]. After 14 days, their drought tolerance was assessed by withholding water for 20 days.

### Relative Water Content and Water Loss Assay

Relative water content (RWC) was measured as described by Thirumalaikumar et al., 2018. Briefly, second true leaves were harvested and their fresh weights (FW) were determined. Leaves were then immersed in distilled water and incubated overnight at 4°C to obtain the saturated weight (SW). Next, leaves were dried at 70°C for 48 h to measure the dry weight (DW). RWC was calculated as % by the formula (FW-DW)/(SW-DW) x 100. RWC was measured in 15 biological replicates per genotype per treatment.

For water loss assays, second true leaves were detached and immediately weighted (FW). Leaves were then placed on filter paper in a growth chamber at 22°C and the weight measured after 1, 2, 3, 4, 5 and 6 h. Water loss was measured in 15 biological replicates each.

### Expression Profiling

Total RNA extraction, cDNA synthesis, and qRT-PCR were performed as described by Balazadeh et al., 2008. The qRT-PCR primers (Supplementary Table S3) were designed using QuantPrime (Arvidsson et al., 2008). ABI-PRISM 7900 HT sequence detection system (Applied Biosystems, Darmstadt, Germany) and SYBR Green (Applied Biosystems, Darmstadt, Germany) were used for qRT-PCR and detection of amplified product.

The transcription pattern of *CaNAC072* and *CaNAC104* was assessed in ten-day-old wild-type pepper seedlings subjected to salinity induced by 120 mM NaCl, drought induced by 10% (w/v) PEG 6000, as well as after treatments with 100 μM ABA over 24 h. Three biological replicates, each a pool of five seedlings, were used for gene expression profiling. The homologue of the Arabidopsis *RD29A* gene, known to be induced by various abiotic stressors (Msanne et al., 2011), i.e., *CaRD29A* (CA.PGAv.1.6.scaffold490.32), was used as a positive control. Data were quantified using the 2^− ΔΔCt^ method based on Ct values of *CaNAC072* and *CaNAC104*, and pepper *ACTIN*, (CA.PGAv.1.6.scaffold786.11) and *GADPH* (TC.CA12g18170) as housekeeping genes.

In the VIGS experiment, the expression pattern of *CaNAC072* (CA.PGAv.1.6.scaffold213.1), *CaNAC104* (CA.PGAv.1.6.scaffold1156.46), *CaNAC019* (CA.PGAv.1.6.scaffold643.12), *CaNAC055* (TC.CA07g18020), *CaDREB2A* (TC.CA05g15500), *CaDELLA* (TC.CA12g02780), and *CaSnRK2* (TC.CA05g02700) was assessed. RNA was extracted from pepper plants infiltrated with TRV2:CaNAC072, TRV2:CaNAC104 and TRV2:00 and subjected to 7 days of water deprivation. Three biological replicates were used for gene expression analyses.

## Results

### Effects of Drought Stress and Recovery on Stomatal Conductance

**FIGURE 1:**
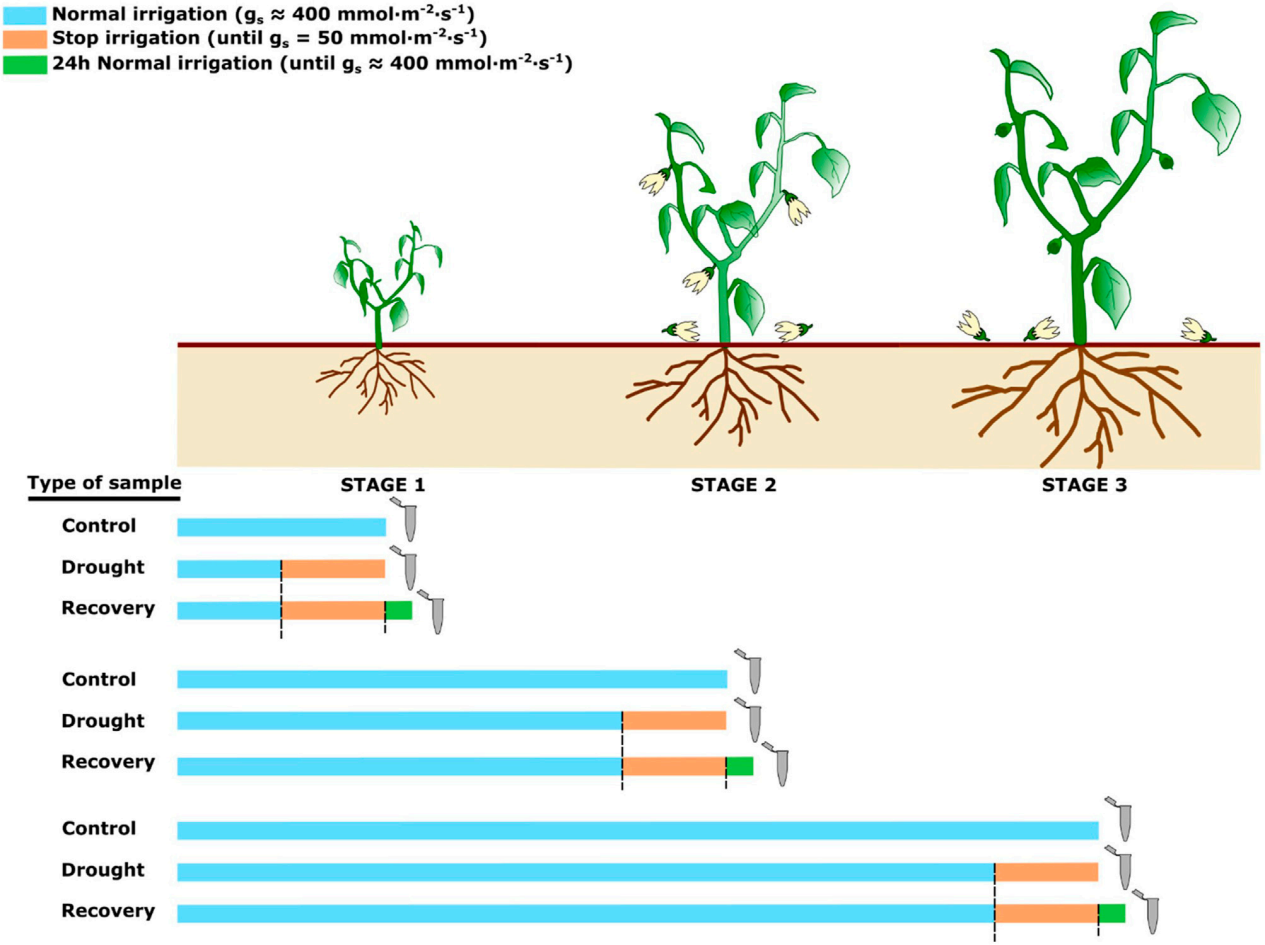
Experimental design of the treatments for control, drought and recovery plants. Grey micro test tubes indicate when the samples were collected. The colour of the bars represents the irrigation status of the plants (light blue = normal irrigation; orange = no irrigation; green = irrigation, 24 h recovery).

We studied the effects of drought stress and recovery (re-watering) on a *C. annuum* inbred line at different plant developmental stages. To this end, plants at the status of five true leaves (stage 1), the third flower (stage 2), and at the setting of the first fruit (stage 3) were exposed to drought stress, by withholding water, and then allowed to recover by re-watering. In the control condition, plants were regularly irrigated. Drought stress was imposed by interruption of irrigation. For recovery experiments, drought-stressed plants were re-watered up to soil water retention (Figure 1). Leaves were sampled after each treatment and used for RNA-seq-based transcriptome profiling.

The effect of water deprivation was assessed through a repeated evaluation of stomatal conductance (g_s_). Control plants showed values of 400 ± 60 mmol m^−2^s^−1^ on average, while drought-stressed plants displayed a progressive decrease of g_s_ until about 50 mmol m^−2^s^−1^ and an evident foliar withering. Afterwards, plants were re-watered, and after 24 h reached a g_s_ value analogous to control plants, and they recovered their leaf turgor.

### RNA-Seq Analyses

RNA-sequencing profiles were obtained from pepper leaves of control, drought-stressed, and drought stressed and then recovered plants at the three developmental stages, as described above. About 33 million paired reads were obtained from each sample. After removing low-quality sequences, adapters and possible contaminations, around 30 million reads (PE and singlets) per sample were obtained (Supplementary Table S4). Clean reads were aligned against the CM334 pepper genome (Kim et al., 2017), resulting in approximately 92% mapping reads on average.

Following quality control by PCA analysis, the three replicates for each condition (control, drought and recovery) clustered together. Only one drought stress replicate at fruit stage was found to be separated from the other two replicates. In addition, a clear separation was found between drought stress replicates and control/recovery samples (Supplementary Figure S1).

Using a false discovery rate threshold of 0.05 and an absolute log_2_ fold change ≥1 (adjusted *p* ≤ 0.01) as the significance cutoff, the expression of 3,680, 3,976 and 1,282 DEGs was found to be significantly regulated by comparing control to drought-stressed plants at stage 1, 2 and 3, respectively. By comparing drought-stressed and recovered plants a whole of 4,155, 2010, 1,328 DEGs were found to be significantly regulated at stage 1, 2 and 3, respectively. At last, when recovered plants were compared with control plants 1,285, 271 and 56 genes where differentially expressed at stages 1, 2 and 3, respectively (Supplementary Table S5).

### Annotation and GO Enrichment of Core Sets of Commonly up- or Downregulated Genes

**FIGURE 2:**
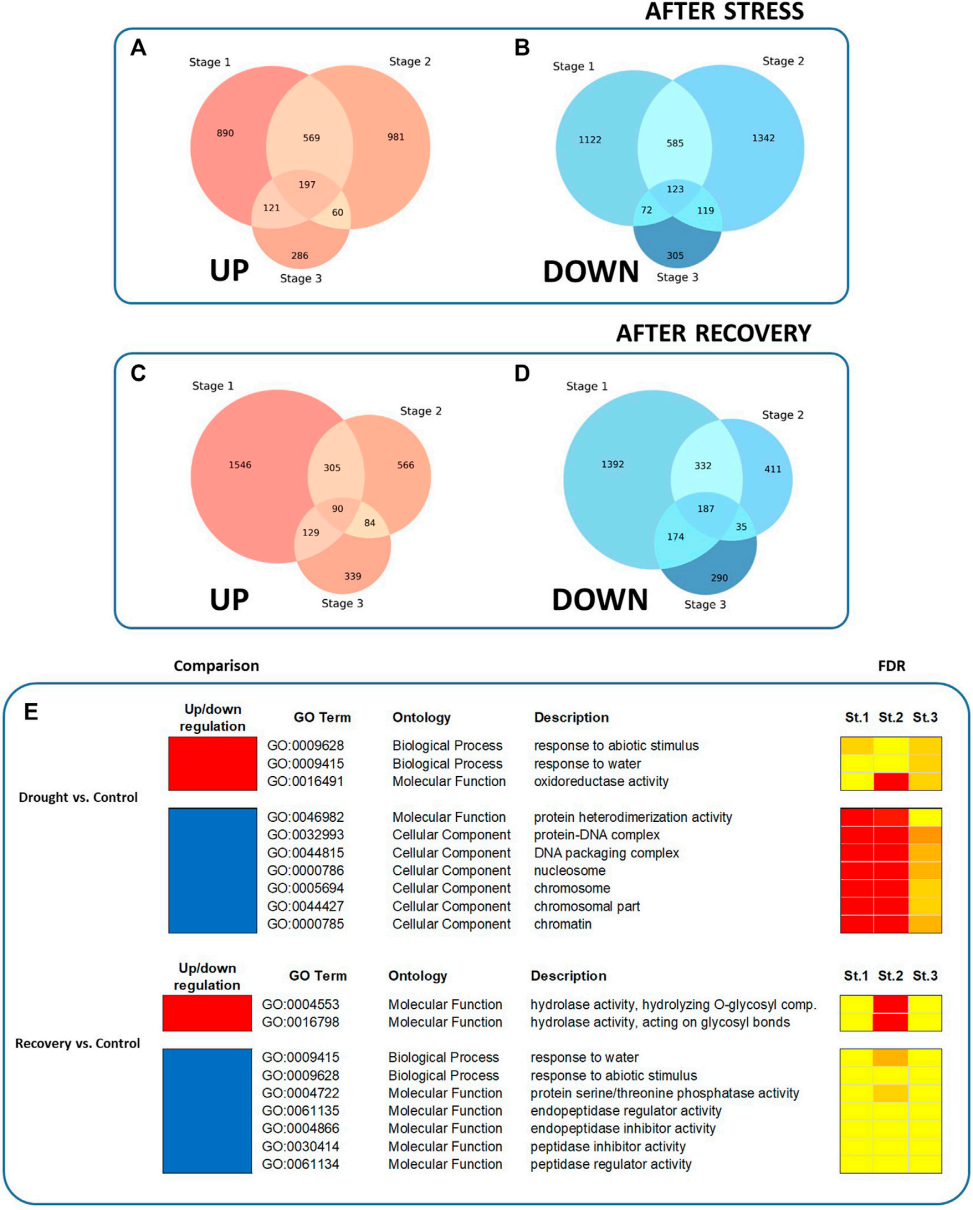
Venn diagram showing intersections of up- or downregulated DEGs, and enriched GO terms, among the DEGs in response to drought and after recovery. **(A)** Genes upregulated after water stress, compared to control plants. **(B)** Genes downregulated after water stress. **(C)** Genes upregulated after recovery. **(D)** Genes downregulated after recovery. **(E)** Enriched GO terms are shown for the three stages of plant development (i.e., St.1 = five true leaves, St.2 = production of third flower, and St.3 = setting of first fruit). Red color represents upregulated GO terms whereas blue color represents downregulated GO terms. Colors on the FDR columns represent different significance levels (yellow: FDR <0.05; orange: FDR <0.01; red: FDR <0.001.

All DEG sets were compared in order to identify groups of commonly regulated genes. At all stages of plant development, a whole of 197 and 123 genes were found to be commonly up- and downregulated after drought, respectively (Figures 2A,B). On the other hand, 90 and 187 genes were found to be commonly up- and downregulated following plant recovery, respectively (Figures 2C,D).

In order to compare the function enrichment of commonly up- or downregulated genes at all stages of plant development, a gene ontology (GO) enrichment analysis was performed. The common over-represented GO terms at all stages are summarized in Figure 2E. Nineteen significant GO terms were identified among all developmental stages. When comparing drought vs control treatments, three enriched GO terms were identified among upregulated DEGs (i.e., GO:0009628, “response to abiotic stimulus”; GO:0009415, “response to water”, and GO:0016491, “oxidoreductase activity”), while seven GO terms were identified among the downregulated DEGs (including e.g. GO:0046982, “protein heterodimerization activity”; GO:0032993, “protein-DNA complex”). On the other hand, when comparing recovery vs drought treatments, two enriched GO terms were identified on upregulated DEGs, i.e., GO:0004553, “hydrolase activity, hydrolyzing O-glycosyl compounds”, and GO:0016798, “hydrolase activity, acting on glycosyl bonds”), while seven were identified among the downregulated DEGs (including e.g., GO:0009415, “response to water”; GO:0009628, “response to abiotic stimulus”). No common enriched GO terms were found by comparing recovery vs. control. All GO terms, including stage-specific enriched GO terms, can be inspected in the Supplementary Tables S6−S17 **.**

**FIGURE 3:**
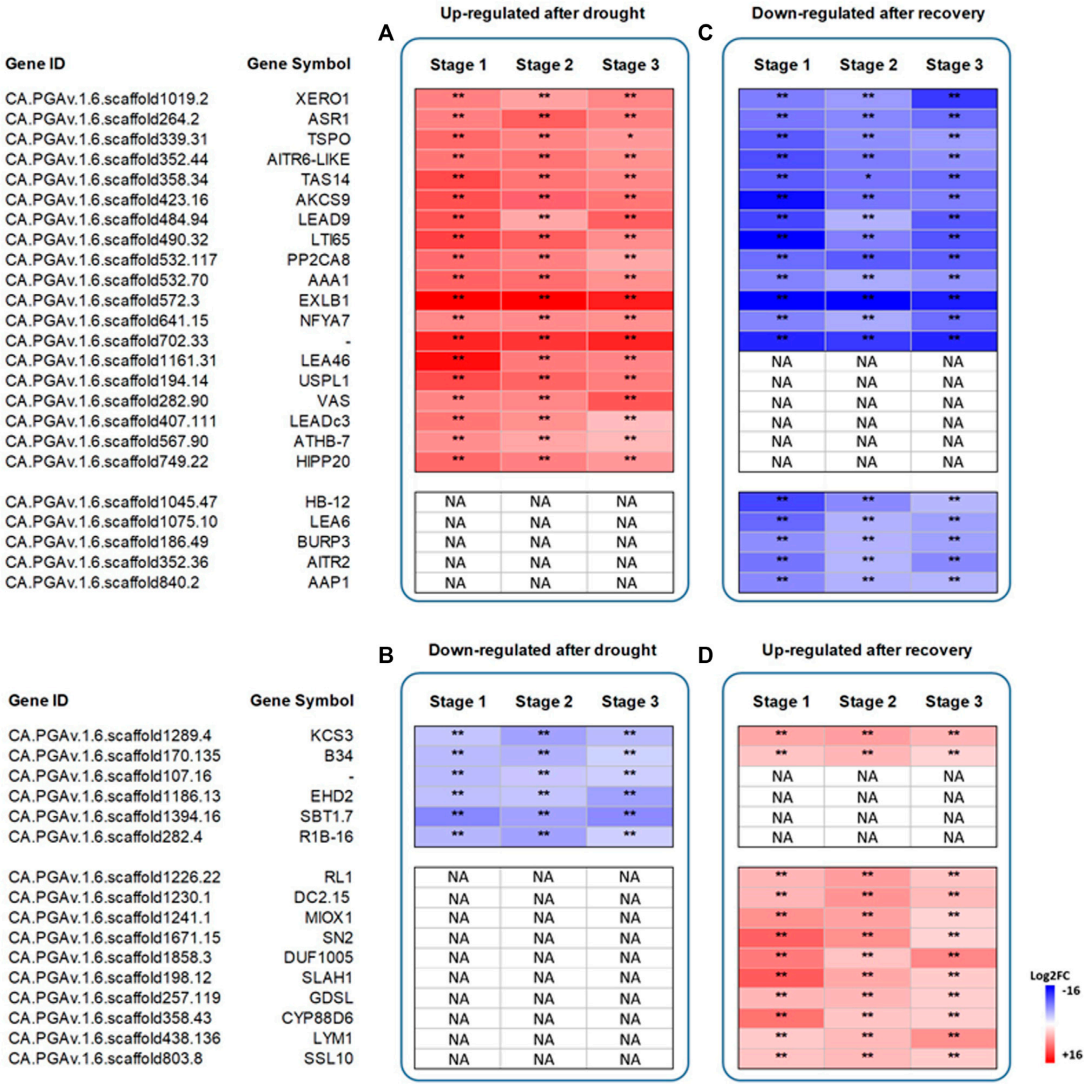
**Heatmap showing the log2 fold change differences in the expression profiles of the top 30 common DEGs at the three stages of plant development.** Genes upregulated after drought stress or recovery (Panel **A** and **D**) and genes downregulated after drought stress or recovery (Panel **B** and **C**). Data represent the means of three biological replicates. Asterisks represent statistically significant differences (*p* values) between control plants and stressed (droughted) plants, and between stressed (droughted) plants and recovery plants. The *p* values were adjusted using the Benjamini and Hochberg method. Corrected *p* value of 0.005 and |log2 (Fold Change)| of 1 were set as the threshold for significantly different expression. White fields indicate the absence of significant differences between treatments. NA stands for “Not applicable”.

Among the 197 commonly up-regulated genes after drought stress, we focused our attention on the 30 most expressed genes at all stages of plant development, and 19 were found to be shared (Figure 3A). Among them are two Late Embryogenesis Abundant protein genes, *LEA46* and *LEA-D29* (CA.PGAv.1.6.scaffold1161.31 and CA.PGAv.1.6.scaffold407.111), several stress-induced genes, such as ABA metabolism-related *ASR1*, *TAS14* and *ATHB-7* (CA.PGAv.1.6.scaffold264.2, CA.PGAv.1.6.scaffold358.34, and CA.PGAv.1.6.scaffold567.90) as well as some plant cell wall-related genes, i.e. *EXLB1, AAA1 KATANIN* (CA.PGAv.1.6.scaffold572.3 and CA.PGAv.1.6.scaffold532.70). Likewise, among the 123 commonly downregulated genes after drought stress, we focused our attention on the 30 most downregulated genes. Six of them were found to be shared and included one gene involved in fatty acid biosynthesis, i.e., *3-KETOACYL-COA SYNTHASE 3* (*KCS3*; CA.PGAv.1.6.scaffold1289.4), and a gene encoding a serine protease (i.e., SBT1.7; CA.PGAv.1.6.scaffold1394.16) (Figure 3B).

After recovery, 12 upregulated genes were found to be in common among the top 30 most upregulated ones. Among them, *MYO-INOSITOL OXYGENASE 1* (*MIOX1*; CA.PGAv.1.6.scaffold1241.1) is involved in providing nucleotide sugars for cell wall polymers, and *3-KETOACYL-COA SYNTHASE 3* (*KCS3*). Lastly, 18 genes were downregulated after recovery, 13 of which were observed to be upregulated after stress. Among them are BURP DOMAIN CONTAINING PROTEIN 3 and homeobox-leucine zipper protein gene ATHB-7 (CA.PGAv.1.6.scaffold186.49 and CA.PGAv.1.6.scaffold1045.47) (Figures 3C,D).

### Differentially Expressed TFs After Drought Stress and Recovery

After drought, the RNA-seq expression profiling revealed 295, 370 and 108 differentially expressed (DE) TFs at stage 1, 2 and 3 of plant development, respectively. Among the ones at stage 1, 162 were up- and 133 downregulated, while at stage 2, 188 were up- and 183 downregulated, and at stage 3, 80 were up- and 28 downregulated (Supplementary Table S18). On the other hand, after the recovery phase a whole of 325, 176 and 106 DE TFs were identified at stage 1, 2 and 3 of plant development, respectively. Of those, at stage 1, 138 were up- and 187 downregulated; at stage 2, 84 were up- and 92 downregulated, while at stage 3, 33 were up- and 73 downregulated (Supplementary Table S18).

Overall, after drought and recovery significant changes occurred in the transcript levels of the five following TF families: MYB, bHLH, ERF, NAC and HSF. Stage-specific TF families were also identified (Supplementary Figure S2). A core set of 31 TF genes were found to be always up regulated after drought and downregulated after recovery at all stages of plant development, among which where MYB, bHLH, CO-like and NF-YA, as well as two NAC TFs (Supplementary Table S19).

### Genome-wide Identification of NAC TFs and Their Expression After Drought and Recovery

**FIGURE 4:**
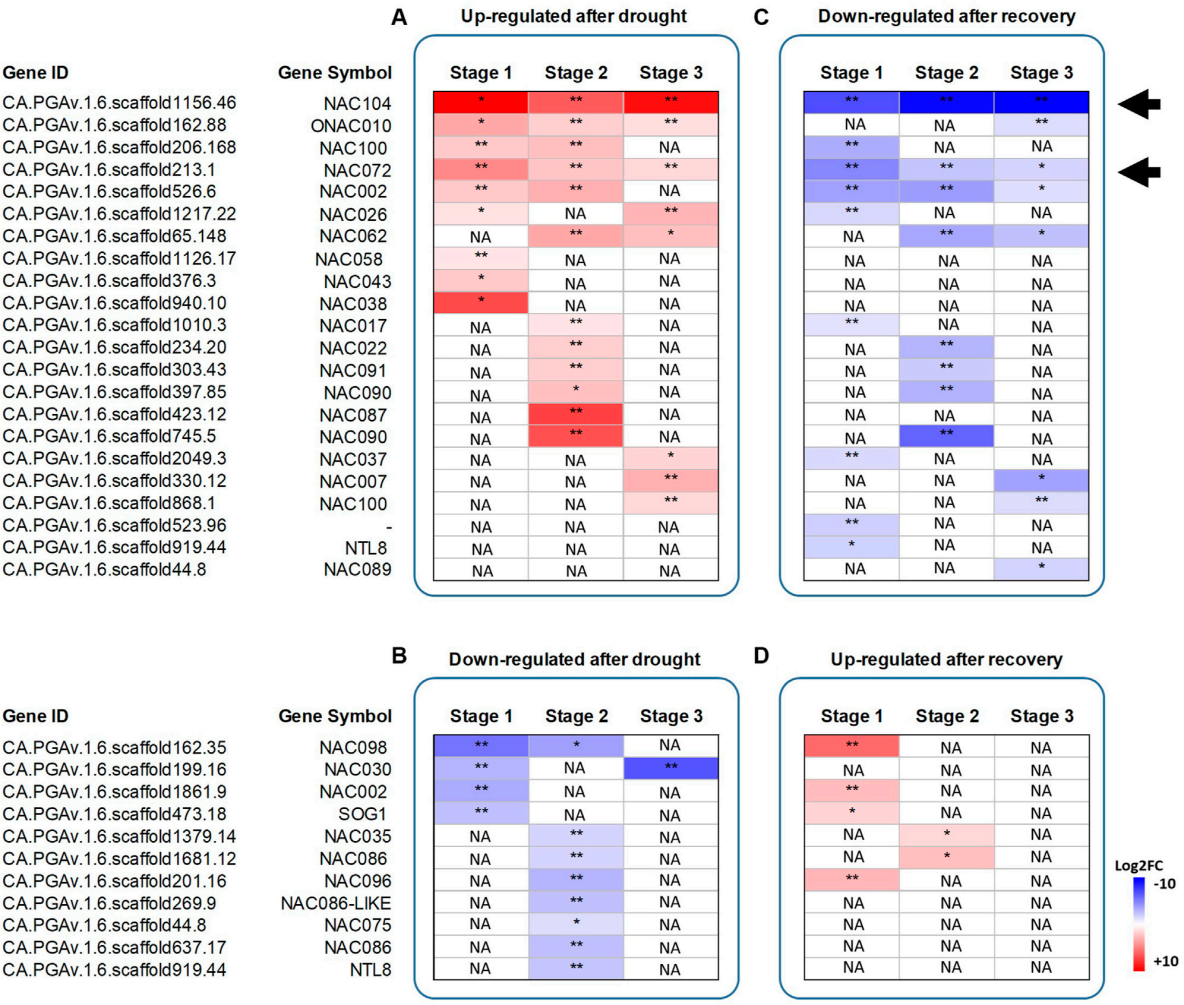
**Heatmap showing the log2 fold change differences in the expression of the differentially expressed NAC TFs identified.** Genes upregulated after drought stress or recovery (Panel **A** and **D**) and genes downregulated after drought stress or recovery (Panel **B** and **C**). Black arrows highlight the two NACs selected for functional characterization, being always upregulated after drought, and downregulated after recovery. Data represent the means of three biological replicates. Asterisks represent statistically significant differences (*p* values < 0.05*, Pvalues < 0.01**) between control and stressed plants, and between drought-stressed and recovered plants. The *p* values were adjusted using the Benjamini and Hochberg method. Corrected *p* value of 0.005 and |log2 (Fold Change)| of 1 were set as the threshold for significantly different expression. White fields indicate the absence of significant differences between treatments. NA stands for “Not applicable”.

We performed a comprehensive analysis to identify NAC genes in the pepper CM334 genome sequence (Kim et al., 2017) on the basis of the NAC domain (PF02365). A whole of 113 NAC TFs were identified (Supplementary Table S20), of which 13, 20 and 9 NACs were differentially expressed after drought stress at stage 1, 2 and 3, respectively (Figures 4A,B). On the other hand, 13, 10, and 8 NAC TFs were found differentially expressed after recovery (Figures 4C,D).

On the basis of our RNA-seq experiment, two members of the NAC TF family, i.e. CA.PGAv.1.6.scaffold213.1 (referred to as *CaNAC072* in the following) and CA.PGAv.1.6.scaffold1156.46 (*CaNAC104*) were found to be always upregulated after drought stress and downregulated after recovery, and thus were selected for functional characterization.

### Subcellular Localization of *CaNAC072* and *CaNAC104*

**FIGURE 5:**
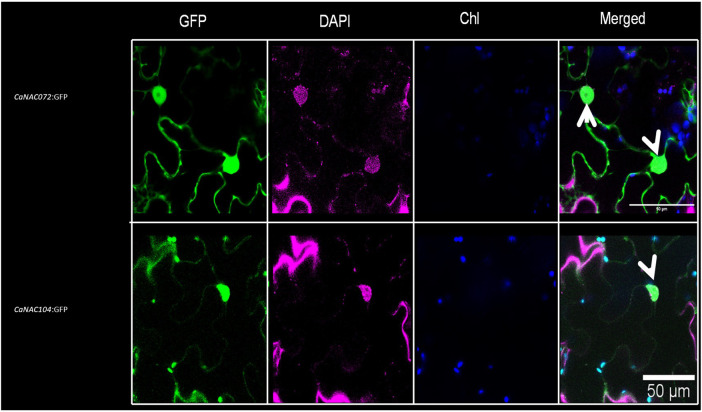
Nuclear localization of CaNAC072 and CaNAC104. Confocal microscopy analysis showing the nuclear localization of CaNAC072:GFP and CaNAC104:GFP upon transient expression in *Nicotiana benthamiana* leaves. Nuclear-specific staining using DAPI confirmed nuclear localization of both TFs. “Merged” indicates the combination of GFP and chlorophyll (Chl) autofluorescence signals. White arrows indicate the nuclear-specific GFP signal. Scale bar, 50 μm.

In-silico prediction analyses of the encoding sequences identified one NLS in CaNAC072 (RKNGSSKLDEWVLCRIYKK) and one in CaNAC104 (KKRK), suggesting a nuclear localization of both proteins. This was confirmed by fusing in-frame the coding sequences of CaNAC072 and CaNAC104, without stop codon, to the green fluorescent protein (GFP) coding sequence under the control of the CaMV 35S promoter. The subcellular localization of each protein was analyzed by confocal laser scanning microscopy after transient expression in *N. benthamiana* leaves. The analysis showed that the GFP signal was localized in the nucleus of leaves transfected with both CaNAC072- and CaNAC104-GFP fusion constructs (Figure 5). This observation is consistent with the role of CaNAC072 and CaNAC104 being transcription factors.

### Effects of Abiotic Stresses on *CaNAC072* and *CaNAC104* Expression

NAC TFs play key roles in plant stress responses. CaNAC072 is the closest homologue of Arabidopsis ANAC072, with an identity of 56.3% and similarity of 67.6% (E value: 5e-132). Lower similarity percentages were detected for the ANAC072 homologues: ANAC019 (identity 53.0%, similarity 64.4%), and ANAC055 (identity 52.8%, similarity 63.1%).

On the other hand, CaNAC104 was found the closest homolog of Arabidopsis ANAC104, with an identity of 40.5% and similarity of 54.8% (E value: 1e-43), the latter being induced by several abiotic stresses and playing a major role in root xylem vessels (Kilian et al., 2007; Tang et al., 2018). Lower similarity percentages were also detected for the ANAC0104 homologues: ANAC02 (identity 5.7%, similarity 10.1%), and ANAC05 (identity 3.8%, similarity 6.6%).

To validate the response of *CaNAC072* and *CaNAC104* to abiotic stresses, we assessed the effects of salinity induced by NaCl, and drought induced by PEG, over 24 h in CCu07 pepper seedlings. Since both salinity and drought stimulate the accumulation of ABA (Marusig and Tombesi, 2020), we also validated gene expression induction upon ABA treatment.

Our treatments were effective in inducing the expression of the stress-related gene *CaRD29A*. The NaCl treatment induced its highest level of expression after 2 h, which progressively decreased in the following hours. Differently, the PEG treatment induced an increase of *CaRD29A* expression after 4 h and a further increase after 6 h, which was followed by a progressive decrease. At last, treatment with ABA caused an increase of *CaRD29A* expression after 2 h onwards following the hormonal treatment (Supplementary Figure S3).

**FIGURE 6:**
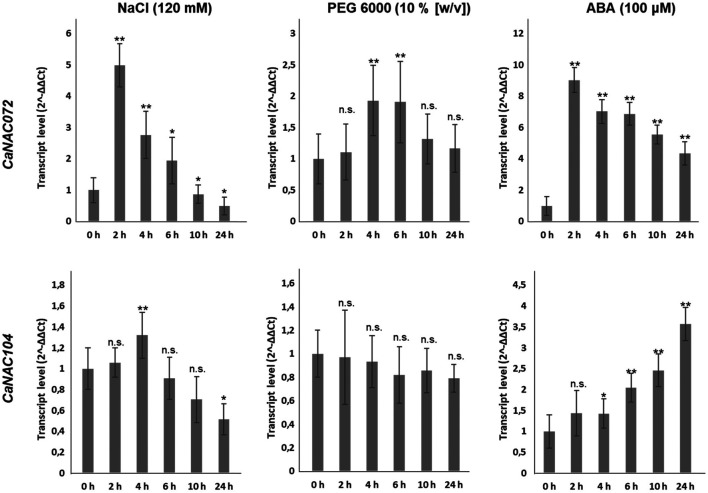
Transcript levels of *CaNAC072* and *CaNAC104* after stress treatments. Gene expression was quantified by qRT-PCR after 0, 2, 4, 6, 10 and 24 h of treatments with NaCl, PEG and ABA. Ten-day-old seedlings were transferred to liquid MS media containing 120 mM NaCl, 10% (w/v) PEG 6000, or 100 μM ABA. Data represent the means ± SD (*n* = 3 biological replicates). Pepper actin and GADPH genes were used as the reference genes. Asterisks (*) indicate statistically significant differences between control condition (0 h of treatment) and stress treatment according to Student’s *t*-test; *, *p* < 0.05; **, *p* < 0.01; n.s. not significant.

As shown in Figure 6, *CaNAC072* transcript levels significantly increased after 2 h of NaCl treatment and gradually decreased in the following hours. The PEG treatment induced the expression of *CaNAC072* only after 4 and 6 h of treatment. Treatment with ABA resulted in a rapid rise (already after 2 h) in the expression of *CaNAC072*. Interestingly, this upregulation was maintained for up to 24 h. In contrast, the NaCl treatment induced the upregulation of *CaNAC104* after 4 h and its transcript level progressively decreased in the following hours, while the PEG treatment did not induce its expression. On the other hand, ABA treatment induced a progressive increase of *CaNAC104* expression from 4 h onwards (Figure 6). These results confirm that *CaNAC072* and *CaNAC104* are differentially induced by salinity and drought and suggest that they are ABA-dependent, with *CaNAC072* being an early-responsive gene and *CaNAC104* acting as a late-responsive gene.

### Effect of Drought Stress on *CaNAC072*- and *CaNAC104*-Silenced Pepper Plants

**FIGURE 7:**
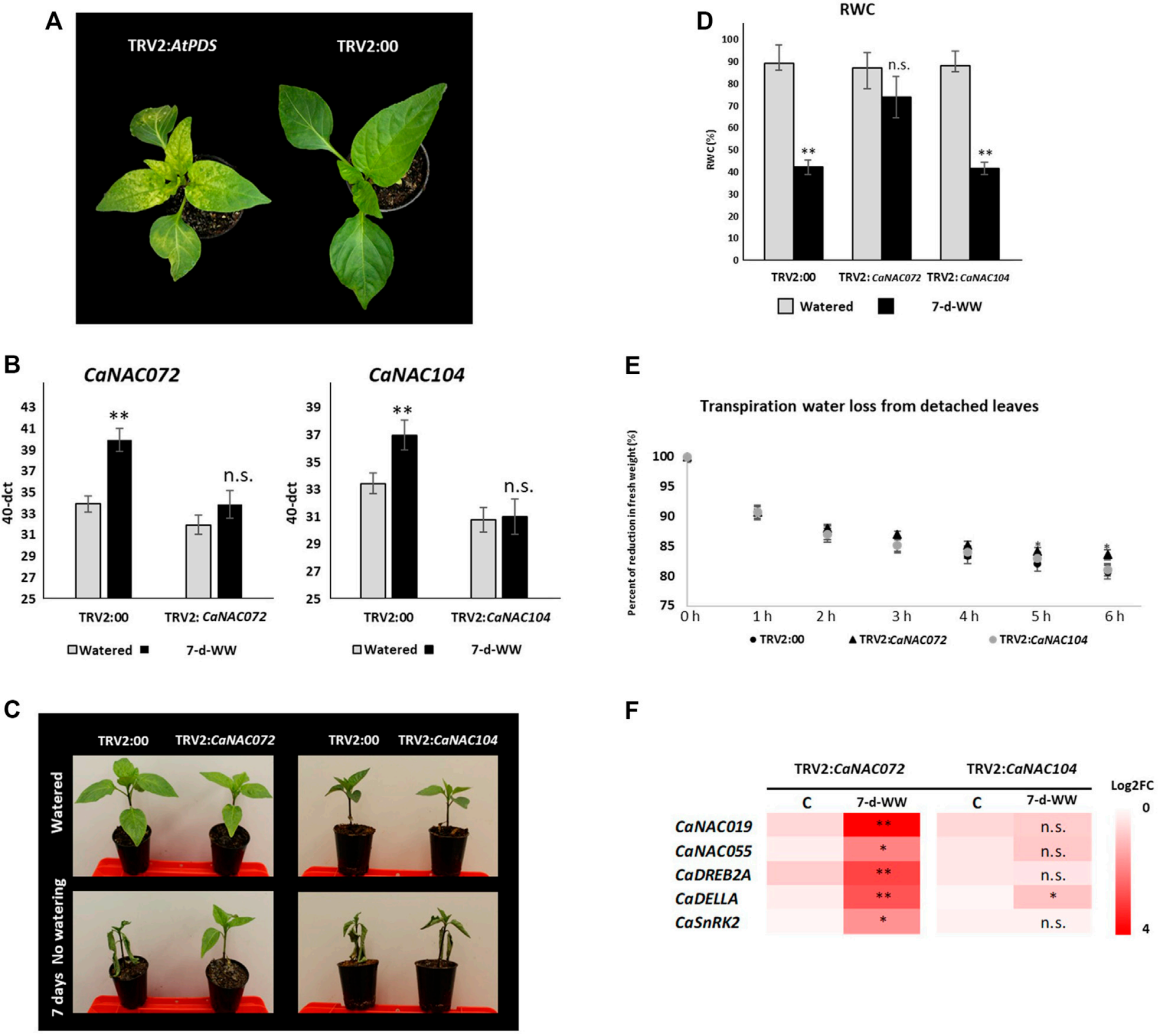
Effect of drought stress on *CaNAC072*- and *CaNAC104*-silenced pepper plants. **(A)** Phenotype of a 25-day-old pepper plant after 12 days of agro-infiltration with TRV2:AtPDS **(left)** and with the empty vector TRV2:00 **(right)**. **(B).** Expression of *CaNAC072* and *CaNAC104* in well-watered control plants (grey bar) and drought-stressed plants (black bar) after 7 days of water withholding (7-days-WW), determined by qRT-PCR. Data represent the means ± SD (*n* = 3 biological replicates). Values are expressed as the difference between an arbitrary value of 40 and dCt, so that high 40-dCt values indicate high gene expression levels. Pepper actin and GADPH genes were used as the reference genes. **(C)** Phenotype of TRV2:00 vis-à-vis with TRV2:CaNAC072 and TRV2:CaNAC104 plants in watering conditions **(upper panel)**, and after 7 days of water withholding **(lower panel)**. **(D)** Relative Water Content (RWC) of TRV2:00, TRV2:CaNAC072 and TRV2:CaNAC104 plants watered (grey bars) and after 7 days of no watering (black bars). Data represent the means ± SD (*n* = 15 biological replicates). **(E)** Trend of leaf fresh weights over 6 h (index of water loss) in detached leaves of TRV2:00, TRV2:CaNAC072 and TRV2:CaNAC104 plants. Data represent means ± SD of 15 biological replicates. **(F)** Heatmap showing the log2 fold change (FC) differences in the expression of five pepper genes (i.e., *CaNAC019*, *CaNAC055*, *CaDREB2A*, *CaDELLA* and *CaSnRK2*) which are orthologues of Arabidopsis drought-responsive genes, in TRV2:CaNAC072, TRV2:CaNAC104, and TRV2:00 plants watered and after 7 days of water withholding. Transcript levels were evaluated by qRT-PCR. Data represent the means of three biological replicates per assay. Asterisks indicate statistically significant differences between *CaNAC072*-non silenced and *CaNAC072*-silenced plants, and between *CaNAC104*-non silenced and *CaNAC104*-silenced plants according to Student’s *t*-test; *p < 0.05, **p < 0.01.

To investigate the role of *CaNAC072* and *CaNAC104* for the response to drought, we reduced their expression through Virus-Induced Gene Silencing (VIGS) in Cuneo CCu07 pepper plantlets. The efficiency of VIGS was confirmed by generating TRV2:AtPDS plants, in which the silencing of the PHYTOENE DESATURASE (PDS) is easily displayed by photo-bleaching of leaves. As expected, plants infected with the empty vector TRV2:00 did not show leaf bleaching (Figure 7A).

*CaNAC072*- and *CaNAC104*-silenced plantlets (TRV2:CaNAC072 and TRV2:CaNAC104, respectively), as well as the ones infected with the empty vector (TRV2:00), were subjected to drought stress (water withholding for 7 days), followed by recovery (rewatering for 7 days). No phenotype alterations were observed between non-infiltrated plants and TRV2:00-infected plants. Similarly, no phenotype alterations were detected between *CaNAC072*- and *CaNAC104*-silenced plants compared to TRV2:00 plants under well-watered conditions. After drought stress, a significant increase in the transcript levels of both *CaNAC072* and *CaNAC104* was detected in plantlets carrying the empty vector TRV2:00, but not in plantlets in which the two NAC TFs were silenced, further confirming the effectiveness of the VIGS (Figure 7B).

After 7 days of drought (water withholding), the TRV2:00 and TRV2:CaNAC104 plantlets showed severe leaf wilting and a marked decrease in their relative leaf water content (RWC), while TRV2:CaNAC072 plants did not, and their RWC was not significantly different from that of well-watered control plants (Figures 7C,D). In order to assess the rate of water loss in silenced plants, detached leaves of TRV2:00, TRV2:CaNAC072 and TRV2:CaNAC104 plants were analysed over 6 h and, as expected, *CaNAC072*-silenced plants showed a better water retention than TRV2:00 and *CaNAC104*-silenced plants (Figure 7E).

We also assessed the survival rate of TRV2:CaNAC072, TRV2:CaNAC104 and TRV2:00 plants after 9 days of water withholding, followed by 7 days of recovery (rewatering) (Supplementary Figure S4). Consistent with their higher RWC and not-withering phenotype, 15.2% of the *CaNAC072*-silenced plants survived, while none of the TRV2:00 and CaNAC104-silenced plants did. This result suggests that CaNAC072 acts as a negative regulator of the drought stress response.

In pepper infiltrated with TRV2:CaNAC072, TRV2:CaNAC104 and TRV2:00, we assessed the transcript profile of five pepper genes orthologous to drought stress-responsive genes in Arabidopsis, i.e. *CaNAC019*, *CaNAC055*, *CaDREB2A*, *CaDELLA* and *CaSnRK2* (Sakuma et al., 2002; Ye et al., 2017). The qRT-PCR analyses revealed that, after 7 days of water withholding, the expression of all of them significantly increased in TRV2:CaNAC072 leaves, while only a bland activation of *CaDELLA* was observed in TRV2:CaNAC104 (Figure 7E). This might indicate a more rapid perception and response to drought stress in *CaNAC072*-silenced plants, which ultimately might explain their improved drought tolerance.

### CaNAC072 Does Not Recover the Function of Its Ortholog in Arabidopsis

*ANAC072* (also known as *RD26*) and its homologs *ANAC019* and *ANAC055* are drought-responsive genes promoting drought stress tolerance in Arabidopsis (Tran et al., 2004). Previous studies showed that the overexpression of either *ANAC019*, *ANAC055* or *ANAC072* confers drought tolerance, while the *anac019/anac055/anac072* triple mutant shows enhanced drought sensitivity (Tran et al., 2004; Ye et al., 2017; Sukiran et al., 2019). In this study, we observed a drought tolerance increase in *CaNAC072*-silenced pepper plants, suggesting a different function in comparison to its homolog *ANAC072* in Arabidopsis. Furthermore, we generated Arabidopsis plants overexpressing *CaNAC072* fused to green fluorescent protein (GFP) (hereafter, CaNAC072:GFP-OX plants) in Col-0 as well as in *anac072* single and *anac019/anac055/anac072* triple mutants. Neither differences in the growth rate nor in the response to severe drought stress were detected between CaNAC072:GFP/Col-0, CaNAC072:GFP/rd26-2, and CaNAC072:GFP/triple mutant plants compared to Col-0, *rd26-2* and the triple mutant (Supplementary Figure S5). Our results thus suggest that CaNAC072 from bell pepper plays a different function than its homolog ANAC072 in Arabidopsis.

## Discussion

The response to drought in plants is a complex and coordinated process involving many biological mechanisms. Here, we subjected *Capsicum annuum* plants of a highly homozygous breeding line to acute water stress until leaf withering, followed by rewatering (recovery phase), at three developmental stages. Following sequencing of RNA extracted from leaves of drought-stressed, recovered and control plants, thousands of DEGs were identified, among which were core sets of commonly and plant development-specific up- and downregulated genes.

Abscisic acid (ABA) is known to act as an endogenous messenger in the regulation of the plant’s water status, and its action can induce stomatal closure, but also systemically signal for adjustment towards severe water shortage (Tuteja, 2007; Todaka et al., 2019). We detected ABA metabolism-related genes downregulated (e.g., *ATHB-7*) or activated (e.g., *ASR1* and *TAS14*) upon drought stress (Godoy et al., 1994; Söderman et al., 1996; Dominguez and Carrari, 2015). Indeed, *TAS14* was found to increase drought and salinity tolerance when overexpressed in transgenic tomato plants, and it was suggested as a promising candidate for enhancing drought tolerance in pepper as well (Muñoz-Mayor et al., 2012).

In plants exposed to drought stress, hydrophilic proteins such as Late Embryogenesis Abundant (LEA) proteins accumulate and cell walls reorganize in order to preserve cell turgor (Shinozaki and Yamaguchi-Shinozaki, 2007; Gong et al., 2010). We detected upregulation of two LEA protein genes: LEA46 and LEA-D29, whose overexpression in Arabidopsis confers tolerance to severe drought (Olvera-Carrillo et al., 2010), as well as of some cell wall-related genes, such as *EXLB1* and *AAA1 KATANIN*, which were upregulated following drought stress and downregulated after recovery. Furthermore, genes such as *MIOX1*, involved in providing nucleotide sugars for cell wall polymers, and *3-KETOACYL-COA SYNTHASE 3* *(KCS3)*, which is required for cuticular wax and root suberin biosynthesis, showed upregulation just after recovery. All in all, our transcriptomic data confirm that cell wall reorganization is a complex cellular process actively involved in the response of C. annuum to water stress and deserves to be better explored for enhancing drought tolerance in the future.

As expected, we identified significant changes in the expression of members of TF families, which are known to be related to stress responses. Among them are bHLH genes involved in the ABA response, HSF genes acting as regulators that prevent the accumulation of damaged proteins, as well as MYBs, ERFs and NACs (Trujillo et al., 2008; Gong et al., 2010); NACs represent one of the largest plant-specific transcription factor gene families (Nakashima et al., 2012; Franco-Zorrilla et al., 2014; Thirumalaikumar et al., 2018).

Previously in *Capsicum annuum* a whole of 104 CaNAC genes were identified by using 47 NAC proteins as query (Diao et al., 2018); here, we identified 113 sequences putatively assigned to the NAC family, presumably due to the different bioinformatics approach adopted. However, to date, only three NACs have been functionally characterized in *C. annuum*: CaNAC035, which acts through multiple signaling pathways and regulates the tolerance to abiotic stress (Zhang et al., 2020); CaNAC064, which positively modulates cold tolerance (Hou et al., 2020); and CaNAC2, which is mainly expressed in seeds and roots, and whose silencing results in increased susceptibility to cold stress (Guo et al., 2015).

On the basis of our RNA sequencing data, only two NACs, i.e., *CaNAC072* and *CaNAC104*, were always upregulated after drought stress and downregulated after recovery, regardless of the stage of plant development; furthermore, following their transient expression in Nicotiana benthamiana leaves, both TFs were localized in the nucleus. Our results thus suggested a possible role of CaNAC072 and CaNAC104 as drought response switchers, and we, therefore, focused our attention on them.

The upregulation of the two NACs was induced at different times following treatments with NaCl, PEG and ABA, whereby *CaNAC072* appeared to be a more rapid responder to abiotic stress than *CaNAC104*. This appears to be confirmed by the not significant upregulation of the slower responsive gene *CaNAC104* after PEG treatment, likely due to a less marked drought stress induced by this treatment compared to the one we imposed (i.e., stop of irrigation up to leaf withering) before our transcriptome analyses.

VIGS represents a fast and powerful reverse genetics tool for analyzing the function of genes in many plant species. In the last decade, VIGS has been successfully applied in Capsicum for the functional characterization of the RING Type E3 ligase CaAIR1, involved in ABA signaling and drought stress response (Park et al., 2014), the ethylene-responsive TF CaAIEF1, involved in enhancing ABA sensitivity and drought tolerance (Hong et al., 2017) and, more recently, the CaNAC035 TF (Zhang et al., 2020). Here, a VIGS protocol, whose efficiency was validated by disabling phytoene desaturase (PDS) as a visible reporter/marker, was applied for transient gene silencing of *CaNAC072* and *CaNAC104*. No difference in the tolerance to drought stress was observed in *CaNAC104*-silenced pepper in comparison to control plants, and a similar relative water content (RWC) and water loss was detected in detached leaves of both plants. This result indicates that the stress-responsive TF CaNAC104 is a component of a complex regulatory network activated by different abiotic stresses, but seemingly it does not play a key role in influencing the response to drought stress. However, considering that CaNAC104 is the ortholog of the Arabidopsis *XND1* gene, which plays a major role in root xylem tracheids (vessels) (Tang et al., 2018), it cannot be excluded that its inhibition by VIGS was not effective and thus did not alter the response of the silenced plants in comparison to the control plants.

Unexpectedly, the silencing of *CaNAC072* improved the tolerance of bell pepper to drought stress, as silenced plants did not display a wilting phenotype after 7 days of no watering, and showed a higher RWC and lower water loss of detached leaves in comparison to control plants. Notably, in contrast to *CaNAC104*-silenced plants, the silencing of *CaNAC072* induced a marked upregulation of stress-responsive genes, including *CaNAC019* and *CaNAC055*. Furthermore, after severe drought (9 days of water withholding), about 15% of the *CaNAC072*-silenced plants survived, while none of the TRV2:00 and *CaNAC104*-silenced plants did.

In Arabidopsis, both ANAC019 and ANAC055 are closely related and functionally redundant homologs of ANAC072, and they act as positive regulators of the drought response (Tran et al., 2004; Ye et al., 2017). On the basis of our RNA-seq experiment, bell pepper plants exposed to acute water stress episodes activated the expression of *CaNAC072*, but not of *CaNAC019* and *CaNAC055* (Supplementary Table S21). However, since the latter two were upregulated in drought-stressed *CaNAC072*-silenced plants, it can be assumed that their upregulation might compensate the disabling of *CaNAC072*, because of a complex gene regulatory network involved in the response to abiotic stresses (Bouché and Bouchez, 2001; El-Brolosy and Stainier, 2017). Similarly, in the Arabidopsis *anac019* mutant, drought stress induced the upregulation of *ANAC055* and *ANAC072* (Sukiran et al., 2019).

*CaNAC072* is the orthologue of the Arabidopsis gene *ANAC072*, a gene activated following drought stress and conferring drought stress tolerance when overexpressed (Tran et al., 2004). With the goal to shed light on its role, we generated transgenic Arabidopsis plants overexpressing *CaNAC072* fused to a green fluorescent protein (GFP) coding sequence (CaNAC072:GFP-OX plants) in Col-0 as well as in *anac072* single and *anac019/anac055/anac072* triple mutants. After 20 days of water withholding, no difference in water stress tolerance between the Col-0 wild type and *CaNAC072* overexpressors was observed. Furthermore, no recovery of function, i.e. increased drought tolerance, was observed when *CaNAC072* was overexpressed in the two Arabidopsis mutant backgrounds. This suggests that the interactions between a NAC protein and the cis-acting elements of target genes as well as the gene regulatory pathway and downstream signaling can vary between plant species. Indeed, previous studies highlighted that orthologous genes might not play the same function in different species, like in the case of Arabidopsis CBF homologs identified in tomato (i.e., LeCBF1, LeCBF2 or LeCBF3) and known to be involved in cold acclimation, and of which only LeCBF1 increased freezing tolerance in transgenic Arabidopsis plants (Zhang et al., 2004).

## Conclusion

Following RNA-sequencing, we identified two transcription factors, CaNAC072 and CaNAC104, that were always up-regulated by acute drought stress and down-regulated after recovery when water was again supplied, at three stages of bell pepper development. The transcriptional regulation and fine-tuning of NAC gene expression is determined by complex signaling pathways: a single NAC gene often responds to several stress factors and participates in the regulation of different processes, as a positive or negative regulator. In our study, the VIGS-mediated silencing of *CaNAC104* did not affect drought tolerance, while silencing of *CaNAC072* increased drought stress tolerance in pepper plantlets. An important result of our study is that *CaNAC072* did not recover the function of its homolog *ANAC072* in *Arabidopsis thaliana*. This observation suggests that the two TF bind to different cis-acting elements of target genes in the two plant species, or that interactions with other TFs or regulatory proteins that ANAC072 might undergo are not faithfully replicated by CaNAC072.

Future research may take advantage of CRISPR/Cas9-mediated genome editing to stably knockout the NAC genes in *C. annuum* for further characterization of their mode of action. This, however, requires the availability of a robust *in vitro* culture and regeneration protocol for this recalcitrant species, which we are currently establishing.

## Data Availability

The datasets presented in this study can be found in online repositories. The names of the repository/repositories and accession number(s) can be found below: National Center for Biotechnology Information (NCBI) BioProject database under accession number PRJNA668245.
