# Supplementary material for: Transcriptome-Based Identification and Functional Characterization of NAC Transcription Factors Responsive to Drought Stress in Capsicum annuum L
Source: Front Genet. 2021 Oct 22;12:743902. doi: 10.3389/fgene.2021.743902 (PMC8570119; doi:10.3389/fgene.2021.743902)
Supplement: Supplementary file 1 [file Presentation1.PPTX]

## Slide 1
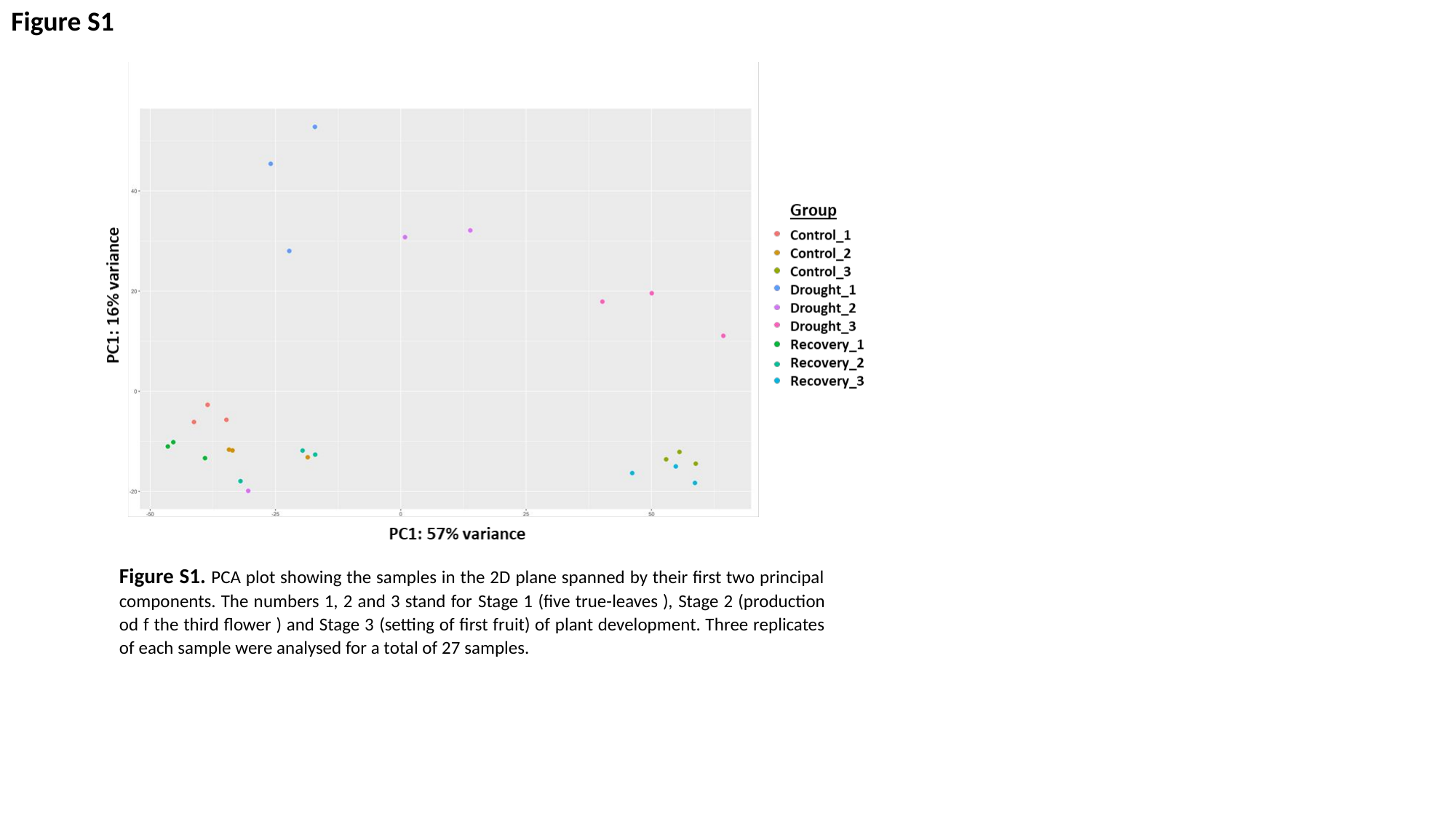

Figure S1
Figure S1. PCA plot showing the samples in the 2D plane spanned by their first two principal components. The numbers 1, 2 and 3 stand for Stage 1 (five true-leaves ), Stage 2 (production od f the third flower ) and Stage 3 (setting of first fruit) of plant development. Three replicates of each sample were analysed for a total of 27 samples.

## Slide 2
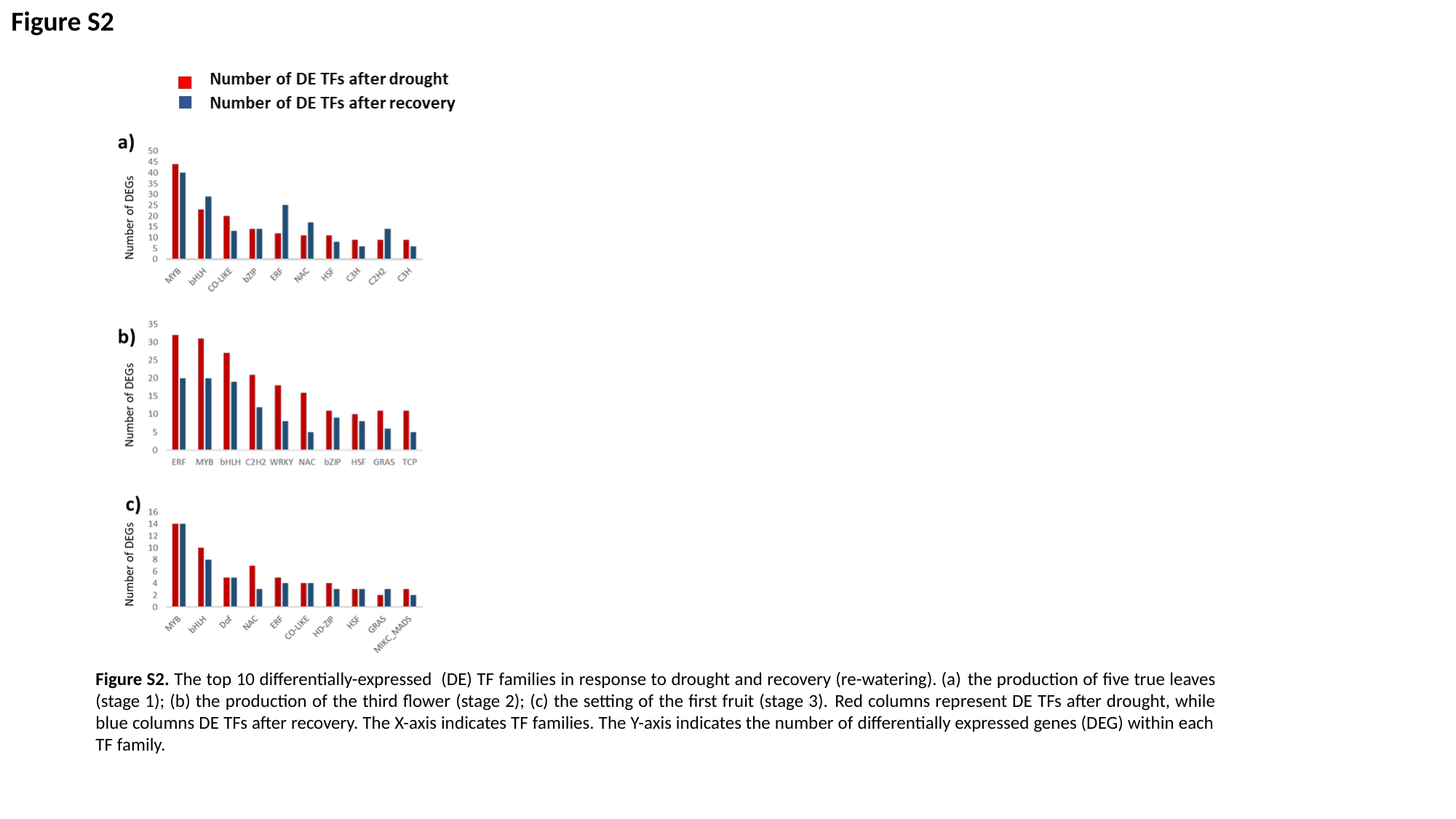

Figure S2
Figure S2. The top 10 differentially-expressed (DE) TF families in response to drought and recovery (re-watering). (a) the production of five true leaves (stage 1); (b) the production of the third flower (stage 2); (c) the setting of the first fruit (stage 3). Red columns represent DE TFs after drought, while blue columns DE TFs after recovery. The X-axis indicates TF families. The Y-axis indicates the number of differentially expressed genes (DEG) within each TF family.

## Slide 3
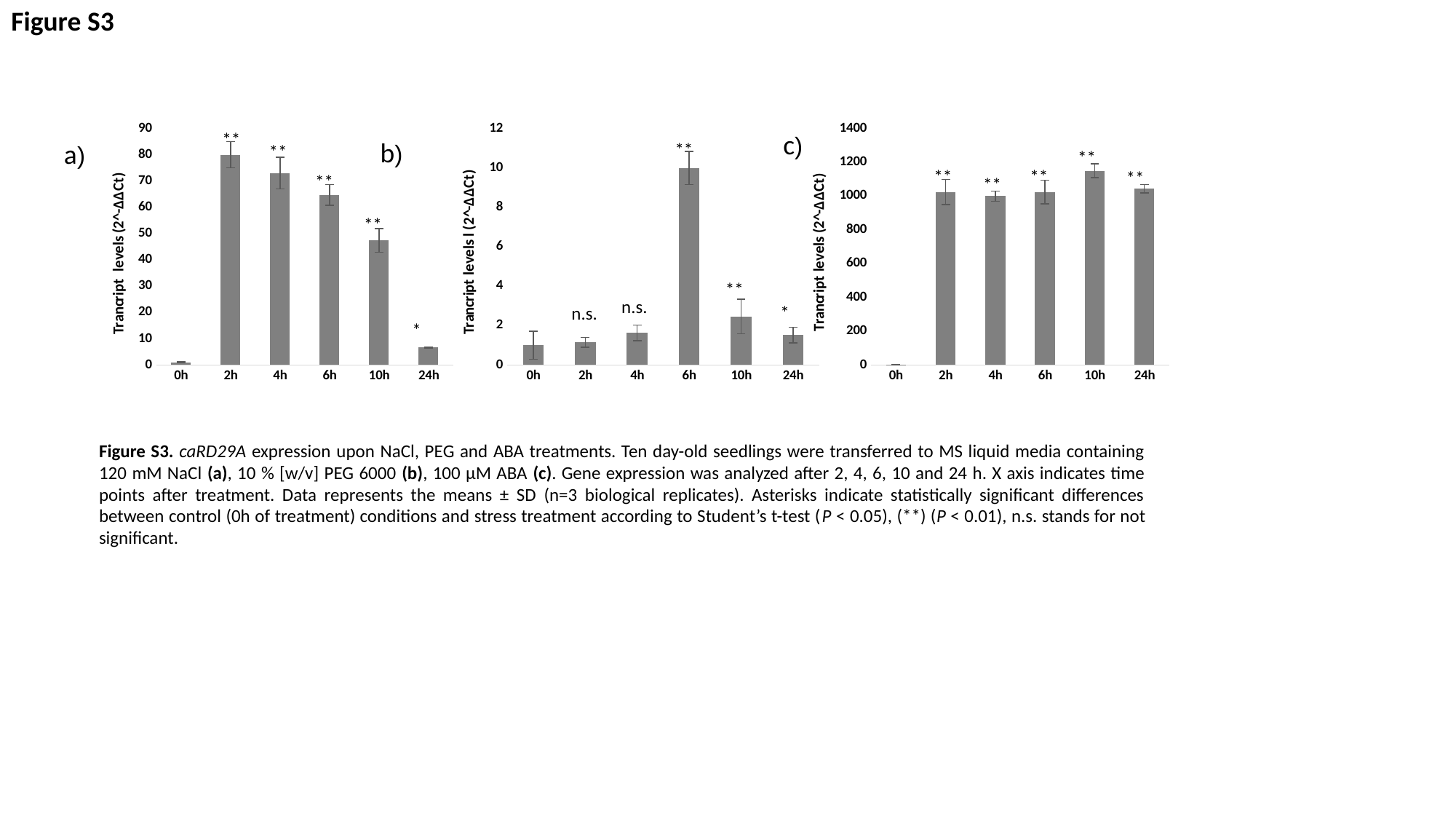

Figure S3
### Chart
| Category | 2^-ddct |
|---|---|
| 0h | 1.0 |
| 2h | 1024.0 |
| 4h | 999.49602563717 |
| 6h | 1024.0 |
| 10h | 1150.26711551697 |
| 24h | 1044.0 |
### Chart
| Category | 2^-ddct |
|---|---|
| 0h | 1.0 |
| 2h | 1.1486983549970344 |
| 4h | 1.6245047927124703 |
| 6h | 9.9960383416998 |
| 10h | 2.4622888266898335 |
| 24h | 1.5157165665103995 |
### Chart
| Category | 2^-ddct |
|---|---|
| 0h | 1.0 |
| 2h | 80.0 |
| 4h | 73.0 |
| 6h | 64.7152360509518 |
| 10h | 47.3966212270372 |
| 24h | 6.70710678118654 |c)
**
b)
a)
**
**
**
**
**
**
**
**
**
**
n.s.
*
n.s.
*
Figure S3. caRD29A expression upon NaCl, PEG and ABA treatments. Ten day-old seedlings were transferred to MS liquid media containing 120 mM NaCl (a), 10 % [w/v] PEG 6000 (b), 100 μM ABA (c). Gene expression was analyzed after 2, 4, 6, 10 and 24 h. X axis indicates time points after treatment. Data represents the means ± SD (n=3 biological replicates). Asterisks indicate statistically significant differences between control (0h of treatment) conditions and stress treatment according to Student’s t-test (P < 0.05), (**) (P < 0.01), n.s. stands for not significant.

## Slide 4
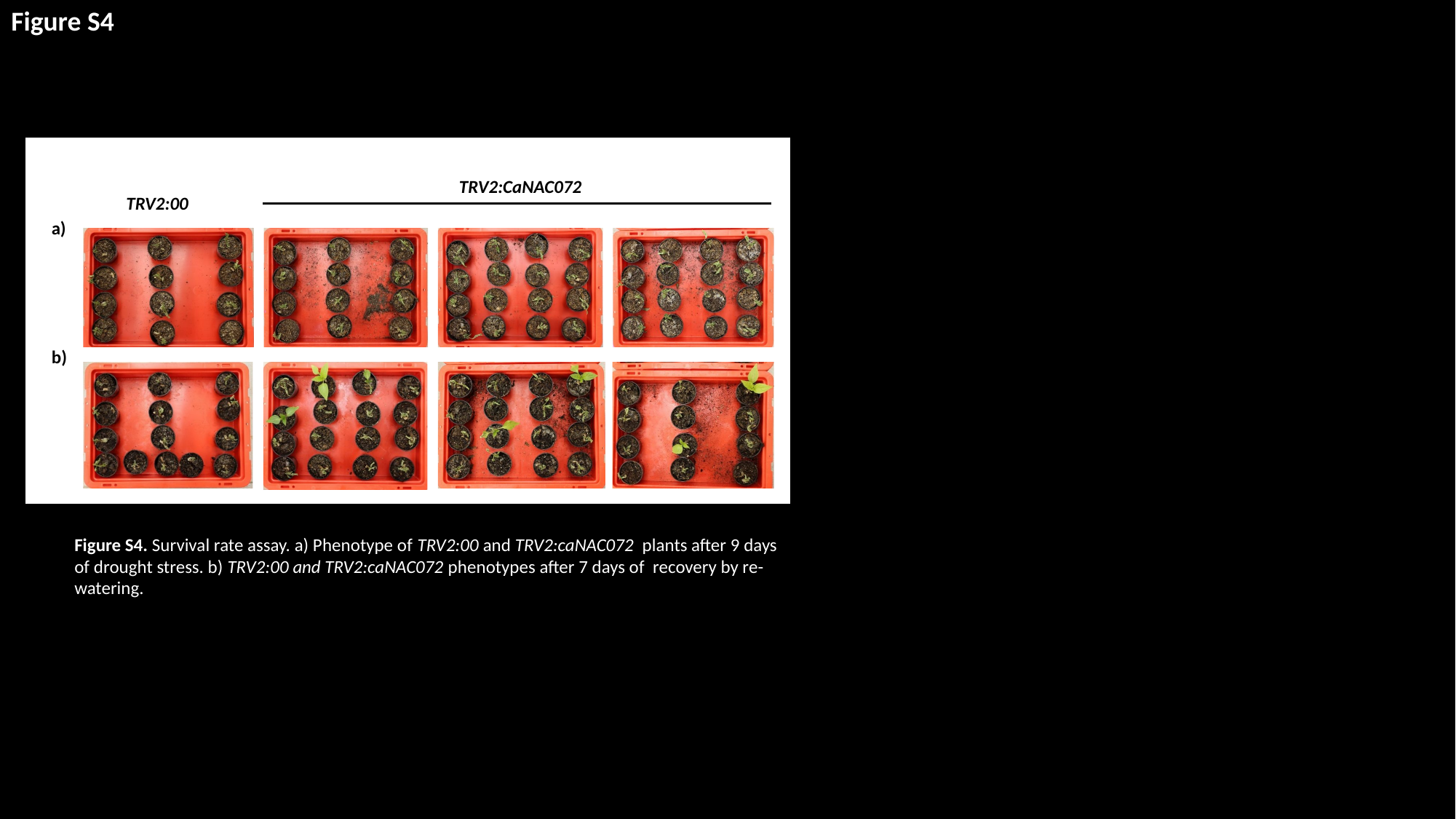

Figure S4
TRV2:CaNAC072
TRV2:00
a)
b)
Figure S4. Survival rate assay. a) Phenotype of TRV2:00 and TRV2:caNAC072 plants after 9 days of drought stress. b) TRV2:00 and TRV2:caNAC072 phenotypes after 7 days of recovery by re-watering.

## Slide 5
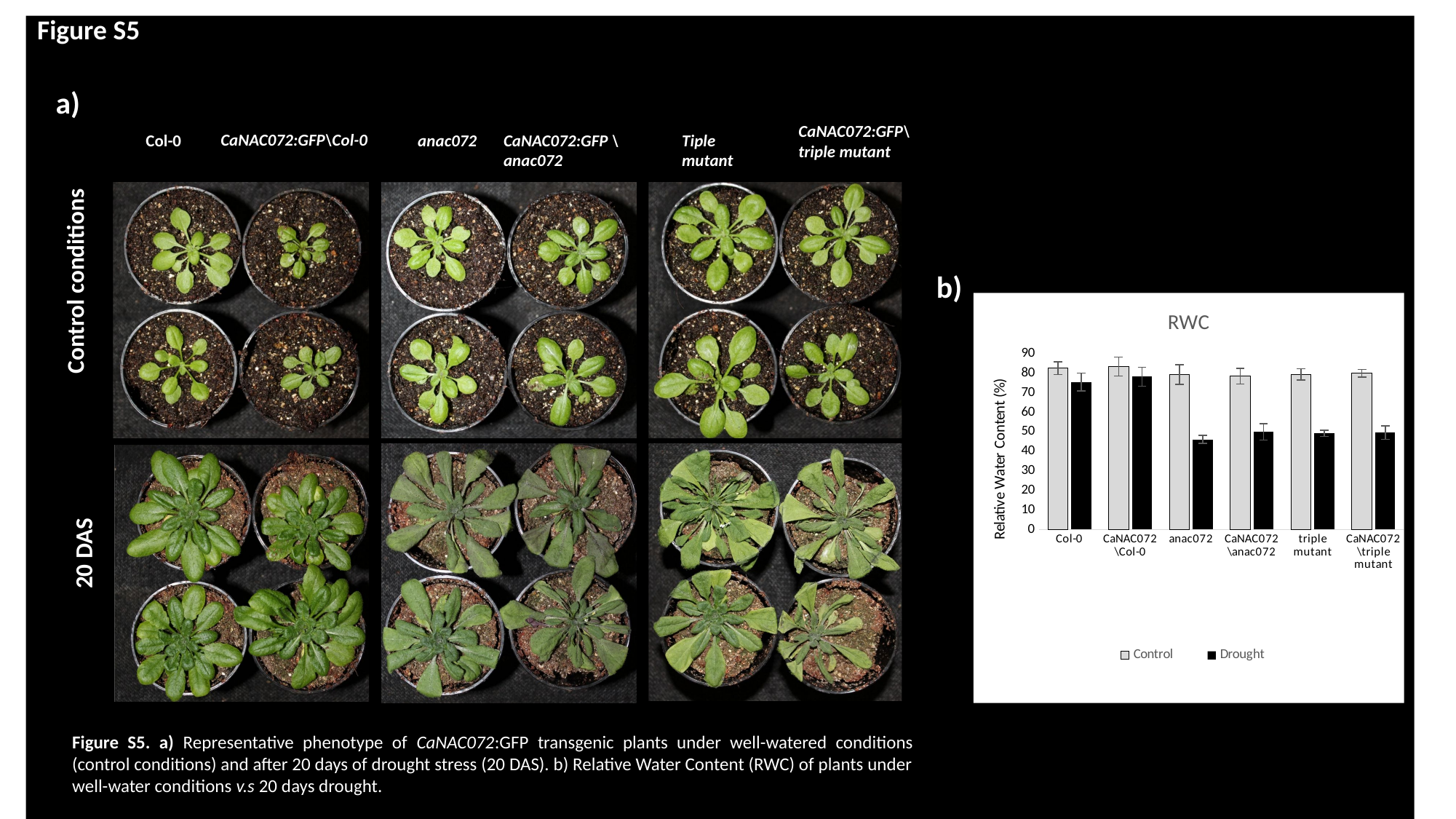

Figure S5
a)
CaNAC072:GFP\
triple mutant
CaNAC072:GFP\Col-0
Col-0
anac072
CaNAC072:GFP \anac072
Tiple mutant
Control conditions
20 DAS
b)
### Chart: RWC
| Category | Control | Drought |
|---|---|---|
| Col-0 | 82.5 | 75.5 |
| CaNAC072\Col-0 | 83.4 | 78.2 |
| anac072 | 79.3 | 46.1 |
| CaNAC072\anac072 | 78.5 | 50.0 |
| triple mutant | 79.4 | 49.2 |
| CaNAC072\triple mutant | 79.9 | 49.6 |caNAC072\col-0
caNAC072\anac072
anac072
caNAC072\triple mutant
Col-0
triple mutant
Figure S5. a) Representative phenotype of CaNAC072:GFP transgenic plants under well-watered conditions (control conditions) and after 20 days of drought stress (20 DAS). b) Relative Water Content (RWC) of plants under well-water conditions v.s 20 days drought.
